# Supplementary material for: A Likelihood Approach for Real-Time Calibration of Stochastic Compartmental Epidemic Models
Source: PLoS Comput Biol. 2017 Jan 17;13(1):e1005257. doi: 10.1371/journal.pcbi.1005257 (PMC5240920; doi:10.1371/journal.pcbi.1005257)
Supplement: S1 File — (TAR.GZ) [file pcbi.1005257.s014.tar.gz › HSPH_Online-SI-Revision/Additions/MSS11/n100-extreme-SEITR/n100-extreme-SEITR_NEW_table0.pdf]

1	R0	{1.38654, 9.85565, 1.}	{2.03307, 8.59564, 1.}	{{2.03307, 8.59564, 1.}, {2.03307, 8.59564, 1.}}	{46.6287, 12.7846, 0.}	inf	18653.	12634.5	{12497.4, 12756.8}	32.2656	0.169207	newly	12085.	8937.24	{8809., 9039.}	26.0468	0.131063	Reff	0.312221	0.687125	{0.683953, 0.689852}	120.077	ar	92379.	80415.8	{80123., 80641.}	12.9501
2	R0	{2.36629, 12.2029, 0.1}	{2.36863, 7.12446, 3.1}	{{2.37035, 7.13655, 3.}, {2.37035, 7.13655, 3.}}	{0.226506, 41.6167, 3000.}	inf	35008.	18750.	{18559.1, 18958.3}	46.4407	0.271184	newly	17453.	15341.5	{15187., 15506.}	12.0981	0.0560167	Reff	0.202507	0.698454	{0.693995, 0.703039}	244.903	ar	99022.	87289.2	{87031., 87483.}	11.8487
3	R0	{2.32253, 12.836, 1.}	{2.14992, 6.52369, 0.1}	{{2.14326, 6.48739, 0.}, {2.14326, 6.48739, 0.}}	{7.59627, 49.1766, 104.}	inf	6387.	16959.4	{16629.4, 17097.1}	53.2772	0.330759	newly	18266.	15795.6	{15562., 15984.}	13.5246	0.0631356	Reff	0.29092	0.822122	{0.817611, 0.826667}	182.594	ar	98997.	83246.	{82839., 83414.}	15.9106
4	R0	{1.79075, 11.2058, 1.}	{2.29496, 8.77947, 0.1}	{{2.2983, 8.81055, 0.}, {2.2983, 8.81055, 0.}}	{28.1563, 21.6524, 100.}	inf	6785.	18855.7	{18704.6, 19066.3}	32.6294	0.171567	newly	16022.	13480.1	{13318., 13621.}	15.8651	0.0750399	Reff	0.341174	0.802502	{0.798211, 0.806487}	135.218	ar	96920.	85927.5	{85648., 86214.}	11.3419
5	R0	{2.26952, 9.62371, 0.1}	{2.49133, 6.2492, 0.}	{{2.49133, 6.2492, 0.}, {2.49133, 6.2492, 0.}}	{9.77351, 35.0646, 100.}	inf	2810.	16523.1	{16375.2, 16661.1}	43.902	0.251063	newly	16920.	13996.	{13848., 14126.}	17.2811	0.0824088	Reff	0.133902	0.539147	{0.535537, 0.542644}	302.644	ar	98881.	88995.6	{88760., 89140.}	9.99732
6	R0	{1.57191, 6.89004, 0.1}	{2.30144, 5.77372, 4.94}	{{2.15896, 5.03398, 5.}, {2.37355, 6.10929, 5.}}	{46.4105, 16.6505, 4840.}	inf	4582.	14839.1	{12515.7, 16191.1}	29.6224	0.155628	newly	17405.	13701.8	{12922., 14253.}	21.2765	0.104202	Reff	0.240125	0.598825	{0.587499, 0.613543}	149.381	ar	95046.	85813.4	{83070., 87406.}	9.71386
7	R0	{1.99226, 17.2339, 1.}	{1.93419, 8.56054, 0.}	{{1.93419, 8.56054, 0.}, {1.93419, 8.56054, 0.}}	{2.91479, 50.3273, 100.}	inf	5638.	11482.7	{11348.9, 11637.5}	61.7537	0.417434	newly	11332.	8287.96	{8177., 8410.}	26.8623	0.135892	Reff	0.270071	0.729853	{0.72682, 0.732382}	170.245	ar	97992.	77455.4	{77176., 77779.}	20.9575
8	R0	{2.63369, 15.1879, 0.1}	{2.40735, 8.12091, 2.}	{{2.40735, 8.12091, 2.}, {2.40735, 8.12091, 2.}}	{8.59421, 46.5304, 1900.}	inf	3741.	19002.1	{18816., 19173.6}	48.3694	0.287105	newly	14766.	13808.2	{13666., 13950.}	6.48646	0.0291403	Reff	0.159786	0.664665	{0.660382, 0.669014}	315.972	ar	99451.	88020.9	{87824., 88241.}	11.4932
9	R0	{2.53271, 9.90552, 0.1}	{2.43398, 5.58375, 2.67}	{{2.41539, 5.51231, 2.}, {2.48443, 5.76577, 5.}}	{3.92689, 43.6299, 2570.}	inf	2374.	15888.6	{15437., 16657.}	49.3363	0.295507	newly	17411.	14601.2	{14367., 14860.}	16.1381	0.0764707	Reff	0.11382	0.543603	{0.535344, 0.54998}	377.599	ar	99355.	88269.2	{87798., 89128.}	11.1578
10	R0	{2.48791, 18.9003, 2.}	{2.10405, 8.58066, 3.}	{{2.10405, 8.58066, 3.}, {2.10405, 8.58066, 3.}}	{15.4291, 54.6004, 50.}	inf	6612.	16589.	{16373.6, 16852.2}	56.8792	0.365336	newly	13614.	12371.3	{12194., 12542.}	9.12818	0.0415976	Reff	0.296509	0.850287	{0.844702, 0.85451}	186.766	ar	99291.	82317.6	{82036., 82597.}	17.0946
11	R0	{2.84414, 18.4765, 1.}	{2.23223, 8.19512, 1.98}	{{2.23156, 8.18891, 2.}, {2.23156, 8.18891, 2.}}	{21.5149, 55.6457, 100.}	inf	5335.	18457.9	{18240.2, 18638.1}	55.996	0.35653	newly	14675.	14100.8	{13927., 14281.}	3.91278	0.0173551	Reff	0.221814	0.817481	{0.813223, 0.821831}	268.543	ar	99654.	85057.6	{84820., 85250.}	14.6471
12	R0	{2.29369, 14.622, 0.1}	{2.34965, 8.72858, 0.36}	{{2.26582, 8.12562, 0.}, {2.49869, 9.80052, 1.}}	{3.99501, 40.3052, 388.}	inf	4308.	17563.	{16281.6, 19699.4}	47.171	0.278793	newly	14159.	12036.9	{11851., 12215.}	14.9874	0.070547	Reff	0.188656	0.661215	{0.651202, 0.667986}	250.487	ar	98982.	86799.4	{85381., 89103.}	12.3079
13	R0	{2.73413, 16.7529, 1.}	{2.5684, 9.75224, 1.}	{{2.5684, 9.75224, 1.}, {2.5684, 9.75224, 1.}}	{6.06141, 41.7878, 0.}	inf	4068.	22294.8	{22106., 22470.3}	42.9172	0.243503	newly	14704.	13990.6	{13837., 14109.}	4.85188	0.0216129	Reff	0.173699	0.698174	{0.693631, 0.702803}	301.944	ar	99543.	90180.	{90024., 90330.}	9.40601
14	R0	{1.79554, 16.5943, 2.}	{1.82266, 8.17412, 4.}	{{1.82266, 8.17412, 4.}, {1.82266, 8.17412, 4.}}	{1.51036, 50.7414, 100.}	inf	7555.	11237.3	{11060.4, 11415.5}	60.2066	0.40022	newly	11642.	8833.4	{8697., 8996.}	24.1247	0.119941	Reff	0.390135	0.843613	{0.840343, 0.846796}	116.236	ar	96821.	73902.6	{73546., 74257.}	23.6709
15	R0	{1.91429, 13.7771, 1.}	{1.96072, 6.67505, 4.}	{{1.96072, 6.67505, 4.}, {1.96072, 6.67505, 4.}}	{2.42521, 51.5497, 300.}	inf	5519.	11618.4	{11424.2, 11810.7}	59.4329	0.391865	newly	13230.	10257.1	{10070., 10441.}	22.4706	0.110576	Reff	0.264038	0.712398	{0.708628, 0.7162}	169.809	ar	97541.	78282.	{77908., 78574.}	19.7445
16	R0	{1.42094, 10.5871, 2.}	{2.93371, 17.1903, 1.}	{{2.93371, 17.1903, 1.}, {2.93371, 17.1903, 1.}}	{106.462, 62.3702, 50.}	inf	7009.	28528.9	{28389., 28659.4}	39.9571	0.145992	newly	12953.	11022.7	{10912., 11147.}	14.9024	0.0700977	Reff	0.417131	0.827896	{0.824024, 0.831969}	98.4738	ar	93044.	93567.	{93461., 93675.}	0.56211
17	R0	{1.45515, 6.16466, 0.1}	{2.16557, 5.46827, 2.74}	{{2.08803, 5.04943, 2.}, {2.19476, 5.61841, 3.}}	{48.8214, 11.2965, 2640.}	inf	6217.	16263.6	{14866.7, 16980.9}	21.591	0.106182	newly	20574.	16955.8	{16573., 17237.}	17.5862	0.0840474	Reff	0.356002	0.761062	{0.754628, 0.766876}	113.78	ar	93254.	83468.3	{81623., 84352.}	10.4936
18	R0	{2.85669, 13.2416, 0.1}	{2.4816, 7.08393, 3.96}	{{2.49502, 7.1476, 4.}, {2.49502, 7.1476, 4.}}	{13.1303, 46.5025, 3860.}	inf	3137.	19856.4	{19879.2, 20213.1}	48.2082	0.286462	newly	17189.	15946.4	{15801., 16148.}	7.22898	0.0326646	Reff	0.132179	0.65065	{0.64595, 0.653579}	392.249	ar	99644.	88980.4	{89005., 89371.}	10.7017
19	R0	{2.56417, 17.9558, 0.1}	{1.9601, 6.67023, 4.}	{{1.96072, 6.67505, 4.}, {1.96072, 6.67505, 4.}}	{23.558, 62.8519, 3900.}	inf	6398.	13854.5	{13626.1, 14065.2}	64.9899	0.455855	newly	14542.	12941.	{12748., 13113.}	11.0098	0.0506875	Reff	0.282084	0.845138	{0.841241, 0.849077}	199.605	ar	99389.	78459.7	{78130., 78789.}	21.058
20	R0	{2.27517, 11.1864, 0.1}	{2.46851, 7.3376, 1.32}	{{2.45236, 7.26016, 1.}, {2.55331, 7.74418, 3.}}	{8.49776, 34.4061, 1220.}	inf	3799.	18421.	{18008.1, 19495.7}	42.3659	0.239489	newly	16864.	14233.	{14056., 14445.}	15.6015	0.0736906	Reff	0.172117	0.614596	{0.607907, 0.620877}	257.081	ar	98894.	88691.3	{88279., 89731.}	10.3168
21	R0	{2.35735, 10.4735, 0.1}	{2.4545, 6.43203, 3.92}	{{2.45956, 6.45922, 4.}, {2.45956, 6.45922, 4.}}	{4.21733, 38.5876, 3820.}	inf	3960.	18783.2	{18652.3, 19038.8}	43.731	0.249842	newly	18543.	16166.5	{16007., 16332.}	12.8159	0.0595783	Reff	0.172039	0.634533	{0.629353, 0.638899}	268.83	ar	99036.	88537.7	{88373., 88836.}	10.6005
22	R0	{1.79551, 11.9179, 2.}	{2.19024, 8.25876, 1.}	{{2.19024, 8.25876, 1.}, {2.19024, 8.25876, 1.}}	{21.9841, 30.7029, 50.}	inf	5574.	15744.6	{15558.7, 15902.3}	41.1	0.229899	newly	14003.	11461.3	{11316., 11580.}	18.151	0.087006	Reff	0.270045	0.692125	{0.687909, 0.695788}	156.3	ar	97033.	84155.1	{83903., 84405.}	13.2716
23	R0	{1.23545, 6.31687, 0.1}	{2.37615, 9.11994, 4.}	{{2.37615, 9.11994, 4.}, {2.37615, 9.11994, 4.}}	{92.331, 44.3743, 3900.}	inf	4400.	17286.4	{17125.4, 17430.5}	23.9789	0.0933368	newly	13260.	11262.4	{11141., 11374.}	15.0647	0.070927	Reff	0.306775	0.61513	{0.611634, 0.618887}	100.515	ar	88739.	87502.9	{87286., 87686.}	1.393
24	R0	{2.24089, 10.1789, 0.1}	{2.77394, 8.24535, 0.}	{{2.77394, 8.24535, 0.}, {2.77394, 8.24535, 0.}}	{23.7874, 18.9956, 100.}	inf	5491.	24803.1	{24570.6, 24984.1}	26.579	0.134189	newly	20012.	17787.2	{17613., 17957.}	11.1175	0.0511948	Reff	0.242442	0.7015	{0.695606, 0.707105}	189.348	ar	98868.	92173.6	{91987., 92350.}	6.77107
25	R0	{1.57298, 10.2607, 0.1}	{2.20429, 9.16468, 0.24}	{{2.22376, 9.35432, 0.}, {2.22376, 9.35432, 0.}}	{40.1347, 10.6818, 328.}	inf	7361.	17881.2	{17970.4, 18391.4}	24.8846	0.125854	newly	15457.	12566.2	{12397., 12759.}	18.7021	0.0899475	Reff	0.403627	0.857408	{0.852712, 0.862161}	112.426	ar	94957.	84171.8	{84309., 84886.}	11.358
26	R0	{1.2154, 8.2576, 0.1}	{2.27199, 11.6669, 0.}	{{2.27199, 11.6669, 0.}, {2.27199, 11.6669, 0.}}	{86.9338, 41.2874, 100.}	inf	5404.	18173.5	{18058.8, 18289.1}	20.514	0.0810316	newly	11730.	9881.11	{9758., 9989.}	15.7621	0.0745125	Reff	0.35796	0.721641	{0.718747, 0.724237}	101.599	ar	88421.	85929.1	{85651., 86130.}	2.81818
27	R0	{2.22841, 18.7983, 2.}	{2.7499, 15.3124, 0.}	{{2.7499, 15.3124, 0.}, {2.7499, 15.3124, 0.}}	{23.4018, 18.5436, 100.}	inf	5503.	25382.8	{25256.5, 25492.5}	25.4696	0.12767	newly	11908.	10653.8	{10537., 10770.}	10.5327	0.0483534	Reff	0.242317	0.688882	{0.685574, 0.692284}	184.289	ar	98756.	92276.1	{92104., 92426.}	6.56155
28	R0	{1.27778, 5.36767, 1.}	{2.18781, 5.83992, 3.99}	{{2.18737, 5.83773, 4.}, {2.18737, 5.83773, 4.}}	{71.2197, 8.79809, 299.}	inf	5294.	15755.8	{15509.5, 15968.9}	4.63747	0.020658	newly	18063.	15298.8	{15083., 15489.}	15.3031	0.0721573	Reff	0.340107	0.714912	{0.710109, 0.71999}	110.202	ar	89843.	83869.7	{83612., 84133.}	6.64859
29	R0	{2.38042, 15.8901, 2.}	{2.19024, 8.25876, 1.}	{{2.19024, 8.25876, 1.}, {2.19024, 8.25876, 1.}}	{7.98945, 48.0258, 50.}	inf	6676.	17916.5	{17707.5, 18116.2}	51.8517	0.317437	newly	15388.	13792.6	{13639., 13955.}	10.3676	0.0475541	Reff	0.307407	0.851664	{0.847107, 0.856191}	177.047	ar	99112.	84132.6	{83815., 84401.}	15.1136
30	R0	{2.53188, 12.7607, 0.1}	{2.18081, 6.20341, 0.12}	{{2.17292, 6.16453, 0.}, {2.17292, 6.16453, 0.}}	{13.8659, 51.3866, 212.}	inf	7079.	18087.4	{17679.7, 18248.}	53.8846	0.33642	newly	19690.	18052.1	{17778., 18344.}	8.31838	0.0377481	Reff	0.319143	0.889932	{0.883724, 0.895835}	178.85	ar	99317.	83951.	{83486., 84117.}	15.4717
31	R0	{1.24482, 10.5471, 1.}	{2.30907, 14.0824, 0.}	{{2.30907, 14.0824, 0.}, {2.30907, 14.0824, 0.}}	{85.4944, 33.5194, 100.}	inf	5495.	18237.	{18114.7, 18341.5}	15.5117	0.06262	newly	9931.	8405.93	{8292., 8511.}	15.3567	0.0724314	Reff	0.364384	0.746246	{0.743694, 0.74901}	104.797	ar	89097.	86133.1	{85886., 86370.}	3.32658
32	R0	{2.30446, 17.9607, 1.}	{2.38758, 11.1331, 1.}	{{2.38758, 11.1331, 1.}, {2.38758, 11.1331, 1.}}	{3.60671, 38.014, 0.}	inf	6256.	20536.2	{20377.4, 20681.6}	42.5657	0.240836	newly	13001.	11842.2	{11728., 11973.}	8.91331	0.0405598	Reff	0.270705	0.793219	{0.789333, 0.796942}	193.02	ar	99028.	87618.	{87424., 87829.}	11.522
33	R0	{2.31579, 11.8236, 2.}	{2.45236, 7.26016, 1.}	{{2.45236, 7.26016, 1.}, {2.45236, 7.26016, 1.}}	{5.89718, 38.5961, 50.}	inf	3005.	16722.2	{16575.2, 16871.3}	44.9257	0.259062	newly	15166.	12781.6	{12641., 12936.}	15.722	0.0743058	Reff	0.14124	0.557783	{0.553926, 0.5613}	294.919	ar	99012.	88554.7	{88347., 88749.}	10.5616
34	R0	{1.6678, 10.6744, 1.}	{2.75598, 12.4651, 1.}	{{2.75598, 12.4651, 1.}, {2.75598, 12.4651, 1.}}	{65.2464, 16.7755, 0.}	inf	4965.	22554.5	{22403.4, 22697.9}	3.41517	0.0150961	newly	13645.	11118.4	{10996., 11239.}	18.5169	0.0889478	Reff	0.259643	0.587865	{0.583868, 0.591773}	126.413	ar	95809.	92080.1	{91942., 92190.}	3.892
35	R0	{1.65476, 7.07559, 0.1}	{2.37355, 6.10929, 5.}	{{2.37355, 6.10929, 5.}, {2.37355, 6.10929, 5.}}	{43.438, 13.6568, 4900.}	inf	5229.	17719.7	{17522.6, 17913.5}	24.7537	0.123534	newly	19217.	15996.3	{15784., 16178.}	16.7596	0.0796836	Reff	0.270404	0.650336	{0.645629, 0.654942}	140.505	ar	95746.	87305.4	{87058., 87524.}	8.81561
36	R0	{2.71807, 18.5959, 0.1}	{2.19024, 8.25876, 1.}	{{2.19024, 8.25876, 1.}, {2.19024, 8.25876, 1.}}	{19.4194, 55.5883, 900.}	inf	7257.	18546.8	{18326.8, 18737.}	55.6975	0.353586	newly	15024.	14672.2	{14482., 14846.}	2.34165	0.0103071	Reff	0.322961	0.929892	{0.925718, 0.9346}	187.927	ar	99540.	84232.4	{83967., 84468.}	15.3784
37	R0	{2.6202, 12.7552, 0.1}	{2.45639, 7.27952, 1.08}	{{2.45236, 7.26016, 1.}, {2.45236, 7.26016, 1.}}	{6.25165, 42.929, 980.}	inf	5145.	21226.7	{20906., 21429.2}	45.7547	0.265692	newly	18951.	17397.2	{17166., 17591.}	8.19899	0.0371707	Reff	0.212734	0.765558	{0.759975, 0.772158}	259.866	ar	99426.	88620.4	{88350., 88815.}	10.868
38	R0	{1.47672, 9.81651, 1.}	{1.96072, 6.67505, 4.}	{{1.96072, 6.67505, 4.}, {1.96072, 6.67505, 4.}}	{32.775, 32.0019, 300.}	inf	5442.	11174.2	{10999.6, 11347.8}	46.3964	0.270838	newly	13535.	9783.42	{9617., 9957.}	27.7176	0.141003	Reff	0.299449	0.693932	{0.69045, 0.697118}	131.736	ar	93815.	78221.5	{77932., 78634.}	16.6215
39	R0	{1.90004, 12.3313, 1.}	{2.22277, 8.52759, 1.34}	{{2.19024, 8.25876, 1.}, {2.28593, 9.04943, 2.}}	{16.9855, 30.846, 34.}	inf	6237.	17264.7	{16580.5, 18434.1}	39.9615	0.222014	newly	15042.	12460.7	{12274., 12657.}	17.1606	0.081788	Reff	0.303056	0.748241	{0.742969, 0.752621}	146.898	ar	97413.	84745.3	{83897., 86058.}	13.0041
40	R0	{2.64218, 18.5237, 2.}	{2.25325, 9.0684, 4.}	{{2.25325, 9.0684, 4.}, {2.25325, 9.0684, 4.}}	{14.7202, 51.0443, 100.}	inf	4328.	17094.6	{16943.2, 17241.7}	54.8539	0.345389	newly	13194.	11575.9	{11449., 11709.}	12.2641	0.0568409	Reff	0.17626	0.715843	{0.712442, 0.719202}	306.129	ar	99474.	85268.3	{85031., 85451.}	14.2809
41	R0	{1.6945, 7.17996, 0.1}	{2.12888, 5.20575, 2.49}	{{2.08803, 5.04943, 2.}, {2.21112, 5.66494, 5.}}	{25.6345, 27.4962, 2390.}	inf	7462.	17016.4	{16161., 18679.2}	35.6681	0.192759	newly	23349.	19095.1	{18640., 19841.}	18.2186	0.0874714	Reff	0.379415	0.835278	{0.826493, 0.844321}	120.149	ar	96332.	82879.9	{81833., 84866.}	13.9643
42	R0	{1.84559, 18.0074, 1.}	{1.96658, 10.2409, 1.}	{{1.96658, 10.2409, 1.}, {1.96658, 10.2409, 1.}}	{6.55583, 43.1297, 0.}	inf	6919.	13776.	{13647.8, 13934.3}	52.0683	0.319391	newly	10541.	8645.21	{8540., 8748.}	17.9849	0.0861304	Reff	0.338389	0.790939	{0.787827, 0.793434}	133.737	ar	97358.	78965.7	{78693., 79186.}	18.8914
43	R0	{2.24965, 17.0361, 2.}	{2.10405, 8.58066, 3.}	{{2.10405, 8.58066, 3.}, {2.10405, 8.58066, 3.}}	{6.47226, 49.6325, 50.}	inf	7490.	16715.1	{16500.6, 16903.9}	52.6644	0.324829	newly	14237.	12535.4	{12358., 12678.}	11.9523	0.0553033	Reff	0.339337	0.866254	{0.862125, 0.870249}	155.278	ar	98770.	82350.2	{82047., 82671.}	16.6243
44	R0	{2.54324, 15.7967, 0.1}	{2.44736, 9.16315, 1.}	{{2.44736, 9.16315, 1.}, {2.44736, 9.16315, 1.}}	{3.76985, 41.9932, 900.}	inf	3895.	19533.	{19363.3, 19689.5}	46.1143	0.268535	newly	14170.	12843.7	{12711., 12979.}	9.3597	0.0426938	Reff	0.167193	0.665315	{0.66146, 0.669428}	297.933	ar	99354.	88525.3	{88324., 88718.}	10.8991
45	R0	{2.54943, 14.8032, 0.1}	{2.59708, 9.39342, 0.}	{{2.59708, 9.39342, 0.}, {2.59708, 9.39342, 0.}}	{1.8691, 36.5447, 100.}	inf	4134.	21789.2	{21615.2, 21978.1}	39.9382	0.22141	newly	14996.	13973.5	{13846., 14093.}	6.81842	0.0306828	Reff	0.179352	0.666354	{0.661403, 0.670787}	271.533	ar	99380.	90404.3	{90183., 90582.}	9.03167
46	R0	{2.37755, 12.6461, 2.}	{2.27423, 6.876, 2.}	{{2.27423, 6.876, 2.}, {2.27423, 6.876, 2.}}	{4.34572, 45.6275, 0.}	inf	5896.	18584.4	{18342., 18827.2}	48.7425	0.290266	newly	17991.	16231.	{16040., 16430.}	9.78261	0.0447309	Reff	0.261673	0.786305	{0.780176, 0.791502}	200.491	ar	99096.	85701.1	{85449., 85922.}	13.5171
47	R0	{1.69279, 11.6636, 1.}	{1.90977, 6.27546, 4.}	{{1.89934, 6.19362, 4.}, {1.96072, 6.67505, 4.}}	{12.8181, 46.1962, 300.}	inf	6849.	12080.3	{11692., 13020.2}	53.1552	0.329655	newly	14547.	11612.7	{11373., 11899.}	20.1714	0.0979065	Reff	0.3499	0.787233	{0.78296, 0.791243}	124.988	ar	96214.	76803.8	{76100., 78359.}	20.174
48	R0	{1.82402, 10.0038, 1.}	{2.22264, 7.15666, 3.28}	{{2.21381, 7.10188, 3.}, {2.2769, 7.49313, 5.}}	{21.8541, 28.4606, 228.}	inf	6918.	17768.	{17410.7, 18556.5}	36.3611	0.196383	newly	17877.	15129.4	{14943., 15296.}	15.3694	0.072494	Reff	0.343281	0.797629	{0.793243, 0.80214}	132.355	ar	97076.	84675.3	{84282., 85605.}	12.7742
49	R0	{1.6424, 11.1667, 2.}	{2.53075, 11.6597, 0.}	{{2.53075, 11.6597, 0.}, {2.53075, 11.6597, 0.}}	{54.0885, 4.41504, 100.}	inf	4677.	18964.2	{18831.8, 19116.4}	16.099	0.0762389	newly	12499.	9956.03	{9843., 10062.}	20.3454	0.0988056	Reff	0.249793	0.595355	{0.591411, 0.598294}	138.34	ar	95546.	89551.3	{89395., 89716.}	6.27412
50	R0	{2.05638, 14.3048, 1.}	{2.68687, 12.2271, 1.}	{{2.68687, 12.2271, 1.}, {2.68687, 12.2271, 1.}}	{30.6601, 14.5244, 0.}	inf	6276.	24527.5	{24350.8, 24677.6}	22.3618	0.10993	newly	14245.	12742.8	{12623., 12874.}	10.5454	0.0484098	Reff	0.290299	0.737748	{0.73366, 0.742328}	154.134	ar	98194.	91443.8	{91260., 91589.}	6.87437
